# A randomized comparison of etoposide and cyclophosphamide for stem cell mobilization in newly diagnosed multiple myeloma

**DOI:** 10.1038/s41598-026-46787-1

**Published:** 2026-04-02

**Authors:** Yao Sun, Jieping Li, Yujun Dong, Meng Li, Yueqi Wang, Xilin Chen, Shunzong Yuan, Yun Lu, Yi Ma, Junli Chen, Wen Gao, Wenrong Huang, Yao Liu, Xiubin Xiao

**Affiliations:** 1 Senior Department of Hematology, Chinese People’s Liberation Army General Hospital, 8 East Street, Fengtai District, Beijing, 100071 China; 2 Department of Hematology and Lymphoma, The Affiliated Nanhua Hospital, Hengyang Medical School, University of South China, Hengyang, China; 3 Department of Hematology, Peking University First Hospital, Beijing, China; 4 Department of Hematology, Beijing Chaoyang Hospital, Myeloma Research Center of Beijing, Capital Medical University, Beijing, China; 5 Department of Hematology-Oncology, Affiliated Cancer Hospital of Chongqing University, Chongqing, China

**Keywords:** ​Mobilization, ​Etoposide, ​Cyclophosphamide, ​Multiple Myeloma, ​Autologous Stem Cell Transplantation, Cancer, Medical research, Oncology, Stem cells

## Abstract

Successful autologous stem cell transplantation (ASCT) in newly diagnosed multiple myeloma (NDMM) patients relies on the efficient mobilization of hematopoietic stem cells following induction therapy. While the efficacy of etoposide for stem cell mobilization has been demonstrated in numerous studies, a randomized comparison of the efficacy of cyclophosphamide versus etoposide has previously been lacking. This randomized, open-label, multicenter trial enrolled NDMM patients eligible for ASCT. The inclusion criteria were patients with a diagnosis of NDMM who required stem cell mobilization prior to ASCT. Patients were randomly assigned to receive either high-dose etoposide (VP16; 1.2 g/m^2^) or high-dose cyclophosphamide (CTX; 3.0 g/m^2^) before mobilization. Granulocyte colony-stimulating factor (G-CSF) was administered after chemotherapy to promote stem cell mobilization. The primary endpoint was the proportion of patients achieving CD34 + cell counts ≥ 2 × 10⁶/kg and ≥ 5 × 10⁶/kg. A total of 62 patients were enrolled, with 31 patients in each group. The VP16 group significantly outperformed the CTX group in CD34 + cell collection across all thresholds: ≥2 × 10⁶/kg (100% vs. 77%, *p* = 0.011), ≥ 5 × 10⁶/kg (90% vs. 55%, *p* = 0.002), and ≥ 8 × 10⁶/kg (71% vs. 32.3%, *p* = 0.023). The VP16 group also showed superior success rates in the first apheresis session and achieved higher CD34 + percentages in the collection. Additionally, the VP16 group required fewer apheresis sessions, fewer platelet transfusions, and experienced less nausea during the mobilization period. High-dose etoposide (1.2 g/m^2^) demonstrated superior efficacy and safety compared to high-dose cyclophosphamide (3.0 g/m^2^) for stem cell mobilization in NDMM patients. Based on these findings, etoposide may be considered a more effective and safer option for stem cell mobilization in clinical practice.

The clinical trial was registered on 24/08/2022 (clinical trial identifier NCT05517213).

## Introduction

Multiple myeloma (MM) is the second most common hematological malignancy, accounting for approximately 10% of all hematological cancers, with a median age of onset at 69 years^1^. Despite the advent of novel therapies have significantly improved both disease-free survival and overall survival, autologous stem cell transplantation (ASCT) remains the cornerstone of frontline consolidation therapy^1,2^. The optimal mobilization and collection of peripheral blood stem cells (PBSCs) are critical for the success of ASCT, with the number of CD34 + cells infused being a key determinant of hematopoietic recovery following myeloablation^3^. Research has also demonstrated that higher doses of CD34 + cells are associated with faster neutrophil and platelet engraftment, which further enhances patient outcomes^4,5^. Stem cell mobilization primarily encompasses steady-state mobilization and chemotherapy-based mobilization. In steady-state mobilization, G-CSF monotherapy is often associated with a high mobilization failure rate, necessitating its combination with plerixafor, a selective and reversible antagonist of CXCR4^6^. However, the widespread clinical application of plerixafor in China is limited due to its high cost. In contrast, chemotherapy-based mobilization offers distinct advantages for patients with active disease, providing both antitumor effects and mobilization of hematopoietic progenitor cells (HPCs). Chemotherapy-induced myelosuppression triggers HPC proliferation, significantly increasing the peripheral blood progenitor cell pool compared to baseline and yielding higher CD34 + cell counts than G-CSF monotherapy^7–9^.

Currently, although no standardized chemotherapy-based mobilization protocol exists for MM patients, cyclophosphamide (CTX) has been the most widely used chemotherapeutic agent for stem cell mobilization in patients with MM^10,11^. Etoposide (VP-16) is another commonly utilized chemotherapy agent for stem cell mobilization^12–16^. The early research shows that although the CD34 + cell collection success rate in the VP-16 group is similar to that in the CTX group, the VP-16 group has significantly higher CD34 counts during collection^12^. Retrospective analyses consistently demonstrate that etoposide combined with G-CSF achieves superior mobilization success rates with comparable tolerability relative to cyclophosphamide combined with G-CSF^17,18^. Nevertheless, current guidelines still consider cyclophosphamide combined with G-CSF the preferred mobilizing regimen^19,20^. To the best of our knowledge, a randomized comparison of the efficacy of cyclophosphamide versus etoposide has been lacking, which has resulted in a relatively weak level of evidence. Moreover, existing literature reports that the dosage of etoposide in stem cell mobilization ranges from 375 mg/m^2^ to 2.4 g/m^2^, and the optimal dosage still needs to be further determined through prospective studies^7,16–18,21,22^. Therefore, our center has launched a prospective, multicenter, open-label, randomized controlled trial to compare the mobilization efficacy and safety profiles of the etoposide regimen versus cyclophosphamide in newly diagnosed MM (NDMM) patients eligible for ASCT following first-line induction therapy.

## Patients and methods

### Study design and participants

This prospective, multicenter, randomized, open-label clinical trial was conducted from September 2022 to December 2024 at the Fifth Medical Center of the PLA General Hospital, Affiliated Cancer Hospital of Chongqing University, Beijing Chaoyang Hospital, and Peking University First Hospital. The study was approved by the Ethics Committee of the PLA General Hospital and registered on 24/08/2022 (clinical trial identifier NCT05517213).

Eligible participants were patients aged 18–70 years with NDMM who had received first-line induction therapy and were deemed suitable for ASCT and had expressed willingness to undergo the procedure. Inclusion criteria included an ECOG performance status of 0 or 1, recovery from chemotherapy-related toxicities (peripheral blood leukocytes ≥ 3.0 × 10⁹/L, hemoglobin ≥ 80 g/L, platelets ≥ 80 × 10⁹/L), normal chest CT and ECG findings, and effective contraception for patients of childbearing potential. Female patients were required to be non-pregnant. Exclusion criteria comprised ineligibility for transplantation, grade ≥ 3 cardiopulmonary insufficiency or severe renal disease, active infection (including fever > 37.5 °C of unknown origin), liver function abnormalities (aspartate aminotransferase or alanine aminotransferase ≥ 2× upper limit of normal, total bilirubin ≥ 1.5× upper limit of normal), or a history of severe psychiatric disorders. All participants provided written informed consent.

All enrolled patients were included in the efficacy and safety analyses. Patients who underwent the first ASCT were included in the post-transplant hematopoietic reconstitution assessment.

### Endpoint

The primary endpoint is to compare the proportion of patients achieving CD34 + cell yields of ≥ 2 × 10⁶/kg and ≥ 5 × 10⁶/kg after up to three apheresis sessions following stem cell mobilization in VP-16 group and CTX group. The secondary endpoints included the total number of CD34 + cells collected for double transplant (≥ 8 × 10⁶/kg), the proportion of patients achieving different yield after the first apheresis session, the total number of CD34 + cells collected, the number of apheresis sessions required, the number of times plerixafor was used, and the incidence of hematologic and non-hematologic toxicities.

### Sample size and power analysis

Based on prior literature and a preliminary analysis of our historical cohort, we estimated that the success rate of achieving CD34 + cell counts of ≥ 5 × 10⁶/kg after a maximum of three apheresis sessions would be approximately 90% in the VP-16 group and 60% in the CTX group. A superiority design was employed for the trial, with a significance level set at α = 0.025 (one-sided) and a power of 1 - β = 0.8. The participant ratio between the two groups was 1:1. The sample size calculation indicated that 29 participants per group would be required. Considering an expected 10% dropout rate, we planned to enroll 33 participants per group, yielding a total sample size of 66 participants.

Participants were randomized 1:1 to the VP-16 or CTX group using block randomization. An independent statistician generated the randomization scheme with SAS 9.4 software, and the list was sealed in opaque envelopes maintained by a designated custodian.

### Randomization and procedure

A block randomization method was used in this study. Eligible participants who provided informed consent were randomly assigned in a 1:1 ratio to either the VP-16 group or the CTX group using a computer-generated block randomization schedule. The block size was randomly selected from the numbers 4, 6, 8, and 10 to ensure a balanced allocation between the groups.

Patients were randomly assigned to receive either etoposide at a dose of 1.2 g/m^2^ via a 24-hour continuous intravenous infusion or cyclophosphamide at 3.0 g/m^2^ via intravenous infusion, both administered as a single dose. Following the recovery of blood counts (defined as stabilization of white blood cell counts after their nadir and platelet counts reaching ≥ 50 × 10⁹/L), G-CSF was initiated at a dose of 10 µg/kg/day via subcutaneous injection. G-CSF was administered daily for four consecutive days, with apheresis commencing on the fifth day and G-CSF continuing until the completion of the apheresis process. Apheresis was performed once daily, with a maximum of three consecutive days. The use of plerixafor was recommended under the following circumstances: if the first CD34 + cell collection was ≤ 2.0 × 10⁶/kg or if the first CD34 + cell collection was ≤ 3.0 × 10⁶/kg but the patient was planned for sequential double transplantation. All other treatment-related procedures were carried out in accordance with institutional protocols and guidelines.

### Definition

CD34 + cell yields were categorized as follows: <2 × 10⁶/kg was defined as collection failure, ≥ 2 × 10⁶/kg as successful collection, ≥ 5 × 10⁶/kg as optimal collection, and ≥ 8 × 10⁶/kg as sufficient for two transplants^23–26^. Engraftment criteria were defined as follows: neutrophil engraftment was achieved on the first day of three consecutive days with an absolute neutrophil count (ANC) ≥ 0.5 × 10⁹/L following post-reinfusion nadir; platelet engraftment was achieved on the first day of seven consecutive days with a platelet count ≥ 20 × 10⁹/L without platelet transfusion. Delayed hematopoietic reconstitution was defined as engraftment occurring beyond 28 days post PBSCs reinfusion. Safety and tolerability were evaluated during whole mobilization by monitoring the vital signs, physical examination and laboratory parameters. Adverse events (AEs) were graded according to the National Cancer Institute Common Terminology Criteria for Adverse Events version 5.0. Diagnostic, classification, staging, and response criteria adhered to the Chinese Guidelines^27^.

### Statistical analysis

All statistical analyses were conducted using SPSS version 26.0. Continuous variables were compared between groups using the independent samples t-test for normally distributed data or the Mann-Whitney U test for non-normally distributed data. Categorical variables, encompassing binary and nominal data, were analyzed using the chi-square test or Fisher’s exact test, depending on expected cell frequencies. Ordinal variables were compared using the Mann-Whitney U test. A p-value of less than 0.05 was considered indicative of statistical significance.

## Results

### Patient clinical characteristics

**Fig. 1:**
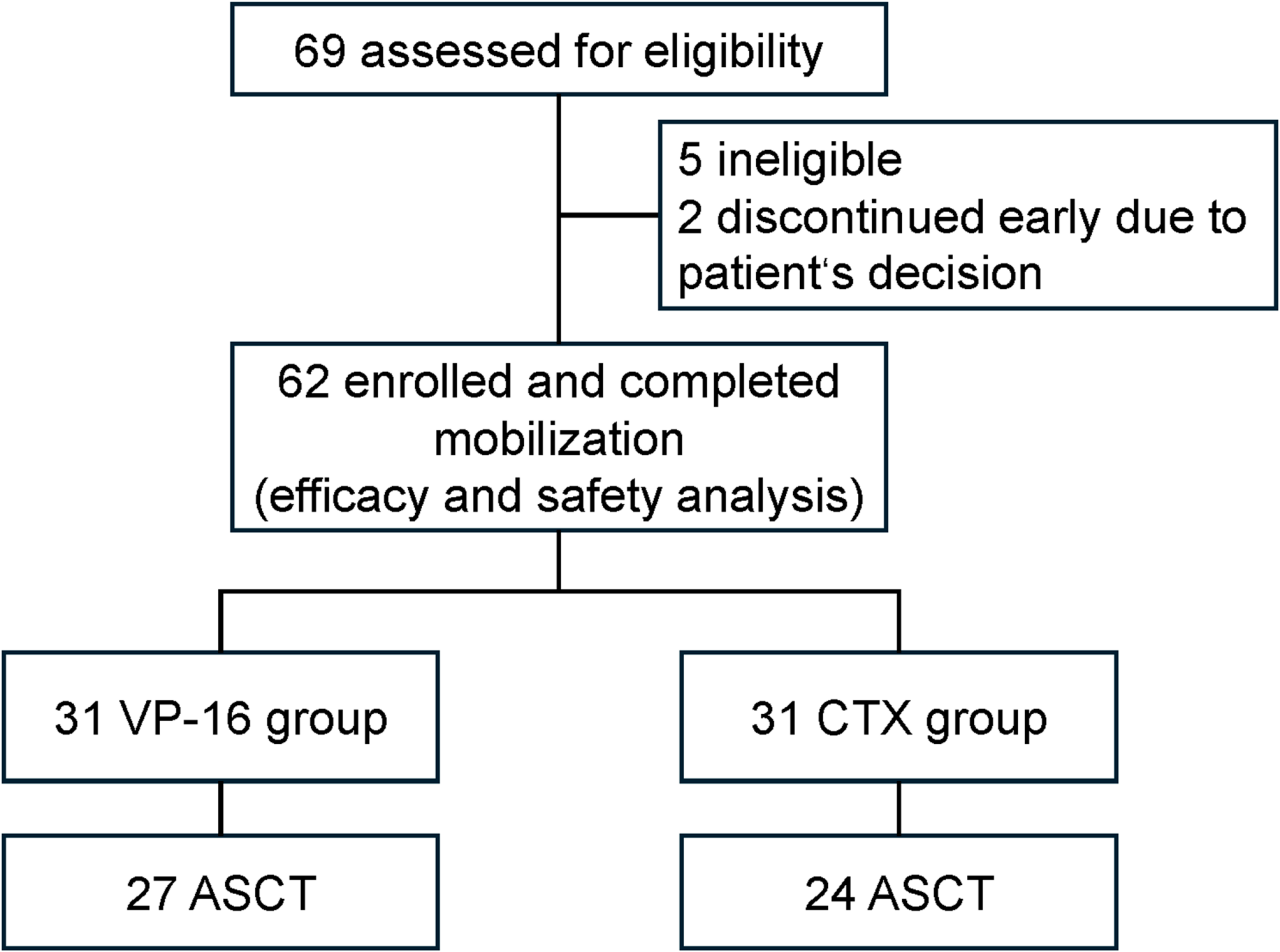
Patient disposition. This flowchart illustrates the enrollment and intervention allocation of participants in the study. Abbreviations: ​*ASCT* autologous stem cell transplantation, ​*CTX* cyclophosphamide, ​*VP-16* etoposide.

Detailed patient disposition is provided in Fig. 1. A total of 62 patients with NDMM were enrolled and assigned to either the VP-16 or CTX group (*n* = 31 each). The groups were well-balanced in terms of baseline clinical characteristics, including age, gender, disease subtype, risk stratification, and pre-collection treatment response (Table 1). All patients achieved at least partial response, except for one patient who had stable disease and requested stem cell cryopreservation.

**Table 1:** Baseline clinical characteristics of patients with different chemo-mobilization regimens (*n* = 62).

Variables	Etoposide (*n* = 31)	Cyclophosphamide (*n* = 31)	*P*-value
Median age, year (range)	55 (42–70)	57 (32–67)	0.187
​Percentage of patients aged ≥ 65 years	3.2%	9.7%	0.612
Male, n (%)	20 (64.5)	23 (74.2)	0.409
Ig type, n (%)			0.418
IgG	13 (41.9)	19 (61.3)	
IgA	8 (25.8)	6 (19.4)	
IgD	1 (3.2)	0 (0)	
Light chain only	8 (25.8)	6 (19.4)	
Other	1 (3.2)	0 (0)	
Cytogenetic risk by FISH, n (%)			0.103
Standard risk	10 (35.7)	14 (58.3)	
High risk(smart 3.0)	18 (64.3)	10 (41.7)	
1q21 amplify	14 (45.2)	8 (25.8)	
t(4,14)	4 (12.9)	2 (6.5)	
t(14,20)	0	1 (3.2)	
t(14,16)	2 (6.5)	0	
Del 17p	3 (9.7)	0	
Missing	3 (9.7)	7 (22.6)	
D-S, n (%)			0.735
I	2 (6.5)	3 (9.7)	
II	5 (16.1)	5 (16.1)	
III	24 (77.4)	23 (74.2)	
R-ISS, n (%)			0.051
I	3 (9.7)	7 (22.6)	
II	16 (51.6)	13 (41.9)	
III	10 (32.3)	4 (12.9)	
Missing	2 (6.5)	7 (22.6)	
With extramedullary mass, n (%)	5 (16.1)	5 (16.1)	1.000
Number of Induction Chemotherapy Cycles > 4, n (%)	9 (29.0)	6 (19.4)	0.374
Number of Induction Chemotherapy Cycles, Median (range)	4(3–8)	4(3–7)	0.603
Frontline treatment, n (%)			
VRD/VCRD	25 (80.6)	24 (77.4)	0.167
DVRD/DVD	6(19.4)	7(22.6)	0.798
Disease status at mobilization, n (%)			
SD	1 (3.2)	0 (0)	0.224
PR	3 (9.7)	8 (25.8)	
VGPR	11 (35.5)	11 (35.5)	
≥CR	16 (51.6)	12 (38.7)	

High risk cytogenetics = del(17p), t(4;14), t(14;16) or gain 1q.

​CR: Complete Remission; VGPR: Very Good Partial Remission; PR: Partial Remission; SD: Stable Disease.

### Outcomes of stem cell mobilization and collection

The results of stem cell mobilization and collection are shown in Table 2. The VP-16 regimen demonstrated superior stem cell mobilization and collection efficiency compared to CTX, with a collection success rate (CD34 + cells ≥ 2.0 × 10⁶/kg) of 100% versus 77% (*p* = 0.011) and an optimal collection rate (CD34 + cells ≥ 5.0 × 10⁶/kg) of 90% versus 55% (*p* = 0.002). Outcomes from the first apheresis further highlighted the advantage of VP-16, with a success rate of 93.5% versus 48.4% (*p* < 0.001) and an optimal collection rate of 64.5% versus 19.4% (*p* = 0.002). Additionally, a significantly higher proportion of patients in the VP-16 group achieved a CD34 + cell yield of ≥ 8.0 × 10⁶/kg, either on first-day apheresis or in total (D1: 41.9% vs. 6.5%; Total: 71% vs. 32.3%, *p* = 0.002, *p* = 0.001), which is sufficient for two transplants. The total number of collected CD34 + cells (×10⁶/kg) on first-day apheresis and in total was significantly higher in the VP-16 group (8.01 vs. 2.00, *p* < 0.001 and 12.25 vs. 6.36, *p* < 0.001, respectively).

**Table 2:** Comparison of mobilization and collection outcomes in patients with different chemotherapy regimens (*n* = 62).

Variables	Etoposide (*n* = 31)	Cyclophosphamide (*n* = 31)	*P*-value
≥ 2 × 10^6^ CD34 + cells/kg, n(%)			
D1	29 (93.5)	15 (48.4)	< 0.001
Total	31 (100)	24 (77.4)	0.011
≥ 5 × 10^6^ CD34 + cells/kg, n(%)			
D1	20 (64.5)	6 (19.4)	< 0.001
Total	28 (90.3)	17 (54.8)	0.002
≥ 8 × 10^6^ CD34 + cells/kg, n(%)			
D1	13(41.9%)	2(6.5%)	0.001
Total	22(71.0%)	10(32.3%)	0.002
CD34 + cells collected, ×10^6^/kg, mean ± SD			
D1	8.01 ± 4.70	2.99 ± 3.71	< 0.001
Total	12.25 ± 5.99	6.36 ± 4.98	< 0.001
MNC cells collected, ×10^8^/kg, mean ± SD			
D1	8.17 ± 4.34	8.63 ± 4.00	0.680
Total	17.47 ± 8.29	23.32 ± 12.34	0.044
Days from start of mobilization to first collection [d, M (IQR)]	18 (17 ~ 20)	19 (18 ~ 20)	0.436
CD34 + cell percentage in MNCs, mean ± SD			
D1	1.13 ± 0.67	0.41 ± 0.63	< 0.001
Total	0.93 ± 0.64	0.26 ± 0.22	< 0.001
Numbers of apheresis			
Mean, n	1.9	2.3	0.020
1, n (%)	6 (19.4)	5 (16.1)	
2, n (%)	21 (67.7)	11 (35.5)	
3, n (%)	4 (12.9)	15 (48.4)	0.002
CD34 + cell percentage in MNCs, mean ± SD			
D1	1.13 ± 0.67	0.41 ± 0.63	< 0.001
Total	0.93 ± 0.64	0.26 ± 0.22	< 0.001
Application of Plerixafor	2 (6.5)	10 (32.3)	0.010

The median time from the initiation of mobilization to the first apheresis was comparable between the two groups: 18 days (range: 13–20) for the VP-16 group and 19 days (range: 13–20) for the CTX group (*p* = 0.436). Patients in the VP-16 group required fewer apheresis sessions on average (1.9 vs. 2.3, *p* = 0.020), with a significantly lower incidence of requiring a third collection (12.9% vs. 48.4%, *p* = 0.002). Fewer patients in the VP-16 group required Plerixafor (6.5% vs. 32.3%, *p* = 0.010). The VP-16 group exhibited significantly higher CD34 + cell percentages on both the first-day apheresis and in the total stem cell collection (D1: 1.13 ± 0.67 vs. 0.41 ± 0.63, *p* = 0.001; Total: 0.93 ± 0.64 vs. 0.26 ± 0.22, *p* = 0.001), despite a lower total mononuclear cell yield, which was attributed to fewer apheresis sessions (*p* = 0.044).

### Adverse reactions during mobilization

Both regimens were generally well tolerated during the mobilization and stem cell collection phases (Table 3). Fewer patients in the VP-16 group required platelet transfusions compared to those in the cyclophosphamide group (9.7% vs. 41.9%, *p* = 0.004) and fewer experienced nausea (3.2% vs. 38.7%, *p* < 0.001). Other non-hematologic toxicities such as infections, vomiting, liver and kidney dysfunction, and electrolyte imbalances, were comparable between the two groups.

**Table 3:** Adverse reactions during stem cell mobilization and collection bof patients with different chemo-mobilization regimens. (*n* = 62)

Variables	Etoposide (*n* = 31)	Cyclophosphamide (*n* = 31)	*P*-value
Hematopoietic system			
Platelet transfusion, n(%)	3 (9.7)	13 (41.9)	**0.004**
Red blood cell transfusion, n(%)	3 (9.7)	1 (3.2)	0.612
Durations of ≥G3 neutropenia, Median day (range)	8(0–14)	7(3–13)	0.121
Durations of G4 neutropenia, Median day (range)	6(0–14)	5(1–11)	0.059
Durations of hospitalization, Median day (range)	15(11–22)	15(10–22)	0.39
Non-hematopoietic system			
Infection, n(%)	7 (22.6%)	8 (25.8%)	0.766
febrile neutropenia, n(%)	7 (22.6%)	6 (19.4%)	0.755
Skin and soft tissue infection, n(%)	1(3.2%)	1(3.2%)	1
Pneumonia, n(%)	1(3.2%)	0	1
Gastrointestinal tract infection, n(%)	1(3.2%)	2(6.4%)	1
Bloodstream infection, n(%)	0	1(3.2%)	1
Non-hematopoietic system			
Nausea, n(%)	1 (3.2)	12 (38.7)	**< 0.001**
Vomiting, n(%)	1 (3.2)	3 (9.7)	0.61
Pharyngodynia, n(%)	0	1 (3.2)	1
Liver dysfunction, n(%)	9 (29)	6(19.4)	0.37
Elevated creatinine, n(%)	2 (6.5)	2 (6.5)	1
Cystitis, n(%)	0	1 (3.2)	1
Hypokalemia, n(%)	23 (74.2)	26 (83.9)	0.35
Hypocalcemia, n(%)	13(41.9)	13 (41.9)	1
Hyponatremia, n(%)	2 (6.5)	1 (3.2)	0.35

### Hematopoietic reconstitution after autologous stem cell transplantation

By May 28, 2025, 51 patients (82.3%) had undergone first ASCT. In the etoposide group (*n* = 27), the infusion of CD34 + cells were significantly higher compared to the cyclophosphamide group (*n* = 24), with 7.94 × 10^6^/kg versus 4.62 × 10^6^/kg, respectively (*P* < 0.001). Median neutrophil engraftment occurred at 10 days and platelet engraftment at 11 days in both groups, with no significant differences. (Table 4)

**Table 4:** Hematologic engraftment following high-dose chemotherapy and autologous stem cell transplantation in patients with different chemo-mobilization Regimens (*n* = 51).

Variables	Etoposide (*n* = 27)	Cyclophosphamide (*n* = 24)	*P*-value
CD34 + cells infusion, ×10^6^/kg, Mean (range)	7.94(3.32–19.89)	4.62(1,41-10.79)	**<0.001**
neutrophil engraftment	10 (9 ~ 11.75)	10 (9.5 ~ 11)	0.975
platelet engraftment	11.5 (9 ~ 15.75)	11 (10 ~ 13)	0.846

## Discussion

In the present randomized trial, the VP-16 group demonstrated significantly higher rates of successful stem cell collection (≥ 2 × 10⁶ CD34 + cells/kg), optimal stem cell collection (≥ 5 × 10⁶ CD34 + cells/kg), and double transplant levels (≥ 8 × 10⁶ CD34 + cells/kg) compared to the CTX group, regardless of whether it was the first collection or the total number of collections. Furthermore, the VP-16 group yielded a greater total CD34 + cell count and a higher proportion of CD34 + cells in the collected product, collectively indicating enhanced mobilization efficiency. In contrast, the CTX group required more apheresis sessions and utilized plerixafor more frequently, leading to additional economic burdens, as well as potential physical harm and psychological stress for the patients. Overall, both regimens exhibited comparable safety profiles; however, the VP-16 group had a lower incidence of platelet transfusion requirements and nausea during the mobilization period. Overall, this study demonstrates that etoposide exhibits superior efficacy and safety in stem cell mobilization when compared to cyclophosphamide. To our knowledge, this study represents the first prospective randomized controlled trial to demonstrate its superior mobilization efficacy compared to cyclophosphamide, with potential implications for refining mobilization strategies in clinical practice.

Li et al.^17^ conducted a retrospective analysis comparing the efficacy of a single dose of VP-16 at 1.6 g/m^2^ in combination with G-CSF with CTX at 4 g/m^2^ combined with G-CSF for stem cell mobilization in patients with NDMM. The results demonstrated that the VP-16 group was more effective with a similar safety profile. In contrast to this study, our research employed a lower dose of VP-16 (1.2 g/m^2^) and achieved similar stem cell collection outcomes with a lower incidence of infections, suggesting that 1.2 g/m^2^ might be a more appropriate dose^18^. Another study also conducted a retrospective analysis comparing the efficacy of VP-16 at 0.75 g/m^2^ combined with G-CSF with CTX at 3 g/m^2^ combined with G-CSF for stem cell mobilization in patients with NDMM and similarly demonstrated superior efficacy of VP-16 ^18^. However, in this study, the total dose of VP-16 was reduced to 0.75 g/m^2^, resulting in a total CD34 + stem cell collection of 7.69 × 10^6^ /kg. This suggests that a lower dose may negatively impact mobilization efficacy, making it difficult to collect sufficient stem cells for two transplants. Additionally, a prospective randomized controlled study from Korea confirmed the mobilization advantage of VP-16 at 0.375 g/m^2^ combined with G-CSF versus G-CSF alone, followed by risk-adapted plerixafor for stem cell mobilization in patients with NDMM^16^. In this regimen, 22.2% of patients in the VP-16 group received plerixafor, and the total CD34 + stem cell collection was 9.5 × 10⁶/kg. Compared to the studies mentioned above, our study suggests that VP-16 at 1.2 g/m^2^ may offer better mobilization efficacy while maintaining safety and cost-effectiveness. However, further research is needed to confirm these findings.

Previous studies have indicated that modern agents, such as lenalidomide- and daratumumab-containing induction regimens used as first-line treatment in transplant-eligible patients, may impair stem cell mobilization, often requiring increased use of plerixafor and G-CSF^28–33^. This study highlights the adaptability of the VP-16 regimen in the era of novel therapies, demonstrating its exceptional mobilization efficacy, even in patients who have received daratumumab-containing and lenalidomide-containing regimens. In accordance with current guidelines, it is recommended that sufficient stem cells be collected for two transplants (8–10 × 10⁶ CD34 + cells/kg) in all MM patients to support either tandem or salvage second transplants^25,26^. Thus, VP-16 may be a more effective mobilizing agent for achieving the recommended CD34 + cell yield sufficient for two transplants, especially for high-risk patients. While the 24-hour infusion regimen for etoposide may pose a temporary inconvenience regarding patient mobility, its favorable efficacy and safety profile may offset this logistical aspect in clinical practice.

Both etoposide and cyclophosphamide are associated with a risk of therapy-related secondary neoplasms, albeit through different mechanisms. Etoposide, a topoisomerase II inhibitor, is linked primarily to therapy-related acute myeloid leukemia (t-AML), with risk being dose-dependent and significantly increased at cumulative doses typically exceeding 4–6 g/m^2^^34^. Our regimen utilized a single dose of 1.2 g/m^2^, which is well below this threshold. Cyclophosphamide, an alkylating agent, is more associated with secondary solid tumors, such as bladder cancer^35^. Long-term follow-up of patients receiving mobilization chemotherapy is warranted to fully characterize and compare these risks.

This study has several limitations. First, despite significant differences between the VP-16 and CTX groups, the sample size is relatively small, and larger, multicenter studies are needed to confirm these findings in diverse populations. Second, while chemotherapy-based mobilization improved stem cell yield, it also increased the risk of complications compared to steady-state mobilization. These risks must be carefully weighed against the benefits, and further research is required to optimize patient outcomes. Additionally, the deliberate delay in G-CSF initiation until after hematologic recovery led to a later median time to first apheresis (18–19 days), although the accompanying outpatient management approach helped reduce overall inpatient hospitalization. Future studies should aim to refine the timing of G-CSF administration to better balance mobilization efficiency, procedural logistics, and healthcare resource utilization.

## Conclusion

Given the superior efficacy and safety of high-dose etoposide plus G-CSF regimen to high-dose cyclophosphamide plus G-CSF regimen, etoposide may be considered a preferred option for mobilization in NDMM patients even in the era of novel therapies.

## Data Availability

Data will be made available upon request to the corresponding author (XBX).
